# Perioperative Efficiency of Sugammadex Following Laparoscopic Cholecystectomy in Clinical Practice

**DOI:** 10.31486/toj.22.0064

**Published:** 2022

**Authors:** Christian Lee, Hana Ahsan, Hoon Chae, Danielle M. Esnard, David Broussard, Stuart Hart, Alex Allain, Brittany Bond, Eric Busch, Preya Jhita, Melissa Matte, Robin Stedman, Jacob Lessing, Joseph Koveleskie, Bobby D. Nossaman

**Affiliations:** ^1^ The University of Queensland Medical School, Ochsner Clinical School, New Orleans, LA; ^2^ Department of Internal Medicine, Rowan University School of Osteopathic Medicine, Stratford, NJ; ^3^ Department of Anesthesiology and Perioperative Medicine, Ochsner Clinic Foundation, New Orleans, LA

**Keywords:** *Antagonists and inhibitors*, *neuromuscular blocking agents*, *rocuronium*, *sugammadex*, *therapeutic equivalency*

## Abstract

**Background:** Studies have proposed that the routine use of the modified gamma-cyclodextrin, sugammadex, could provide perioperative time savings. However, these investigations have been limited to small group analyses. The purpose of this study was to test the effectiveness of sugammadex on perioperative times when compared to neostigmine under general clinical practice conditions following rocuronium-induced neuromuscular blockade for laparoscopic cholecystectomy.

**Methods:** Following institutional review board approval, data from 1,611 consecutive surgical records for laparoscopic cholecystectomy were reviewed. Patient characteristics, type of primary neuromuscular blocking reversal agent, operating room (OR) discharge times, and postanesthesia care unit (PACU) recovery times were the measures of interest. Equivalence testing was used to determine the between-group differences of the reversal agents in the two perioperative time periods of interest.

**Results:** OR discharge times averaged 10.9 (95% CI, 10-11.8) minutes for patients administered sugammadex and 8.9 (95% CI, 8.2-9.7) minutes for patients administered neostigmine. PACU recovery times averaged 77.6 (95% CI, 74.1-81.1) minutes for sugammadex and 68.6 (95% CI, 65.9-71.3) minutes for neostigmine. Equivalence testing demonstrated no improvement in the two perioperative times with sugammadex.

**Conclusion:** These results suggest no perioperative time savings with sugammadex when compared to neostigmine following laparoscopic cholecystectomy under general clinical practice conditions.

## INTRODUCTION

Studies^1-5^ have established the clinical efficacy of sugammadex in reversing rocuronium- or vecuronium-induced neuromuscular blockade, with subsequent studies proposing that routine use of the modified gamma-cyclodextrin could provide perioperative time savings.^6,7^ However, studies examining perioperative time savings with sugammadex have been limited to small group analyses,^8-13^ a meta-analysis,^14^ and studies with hypothetical time efficiency models.^15-17^ The purpose of this study was to examine the clinical effectiveness of sugammadex on perioperative time savings when compared to neostigmine under real-world, non-Hawthorne-effect conditions.^18-21^ Equivalence testing was used to compare postoperative time measures obtained with sugammadex to those obtained with neostigmine.^22^ Laparoscopic cholecystectomy was chosen as the surgical procedure of interest as prior studies have shown neuromuscular blockade provides a favorable influence on abdominal working space conditions during laparoscopic surgery.^23-26^

## METHODS

Following institutional review board approval, data from 1,672 consecutive surgical records for laparoscopic cholecystectomy under rocuronium-induced neuromuscular blockade from May 18, 2020, to May 17, 2021 were reviewed. Sixty-one records were not included in this analysis as sugammadex was used to reverse the neostigmine-associated residual neuromuscular blockade observed in those cases. Patient characteristics, type of neuromuscular blocking reversal agents, operating room (OR) discharge, and postanesthesia care unit (PACU) recovery times were the measures of interest.

### Statistics

Categorical variables are presented as counts and percentages with group differences assessed using chi-square tests. Continuous variables with skewed distributions are presented as medians with 25%-75% interquartile range (IQR) with differences between the two groups assessed by the Wilcoxon rank sum test. Arithmetic and geometric means are also presented as measures of central tendency. Overlay plots with autocorrelation statistics were used to measure the degree of nonrandomness in the data sets. Key analyses are expressed with associated 95% CIs when indicated. Cumulative distribution plots were used to compare percent recovery times of the two reversal agent groups.

Equivalence testing (two one-sided tests [TOST] with 90% CI) compared the between-group differences of sugammadex and neostigmine in the two recovery periods.^22^ The minimum range differences for equivalence in OR discharge times were examined at 2 and 3 minutes based upon a reported 2.7-minute improvement with sugammadex in a prior study.^9^ The minimum range differences for equivalence in PACU recovery times were examined at 15 and 20 minutes, similar to a reported 17-minute improvement with sugammadex in a meta-analysis.^14^ Standardized differences were calculated for the patient characteristics at baseline, with absolute values >0.1 suggesting imbalance between the two reversal agent groups.^27^ *P* values for statistical significance of the frequentist tests were set at <0.005.^28^ The statistical program JMP, version 13.2 (SAS Institute) was used for this study.

### Sample Size Calculations

Based upon pilot data obtained from laparoscopic cholecystectomies (n=641) performed before the introduction of sugammadex and not included in these data, the standard deviation for OR discharge times was 8.5 minutes. Assuming a 90% power, a two-sided type I error rate of 0.05, and an allocation ratio of 1, we estimated a minimum of 762 completed medical records would be needed.^29^

## RESULTS

The medical records of 1,611 patients undergoing laparoscopic cholecystectomy during a 1-year period provided a sufficient sample for the study of clinical effectiveness in the measurements of interest.^29^ Patient demographics are shown in the Table. The largest absolute standardized difference in patient characteristics was observed for the American Society of Anesthesiologists (ASA) physical status classification, with a higher percentage of sugammadex used in patients with ASA physical status classifications of III and IV. In this subgroup of patients, median OR discharge times were 9 [IQR, 6-12] minutes for sugammadex and 8 [IQR, 6-11] minutes for neostigmine (chi-square = 3.1, *P* = 0.0879). Median PACU recovery times in this subgroup were 65 [IQR, 42-90] minutes for sugammadex and 64 [IQR, 43-89] minutes for neostigmine (chi-square = 0.04, *P* = 0.8415).

**Table.** Characteristics for 1,611 Patients Receiving Sugammadex or Neostigmine as the Primary Neuromuscular Blocking Reversal Agent Following Laparoscopic Cholecystectomy

Characteristic	Sugammadex, n=601	Neostigmine, n=1,010	Standardized Difference
Age, years, median [IQR]	49 [35-62]	47 [33-61]	–0.05
Male	151 (25.1)	294 (26.7)	–0.07
Weight, kg, median [IQR]	86 [71-103]	87 [74-102]	–0.02
American Society of Anesthesiologists Physical Status Classification			–0.17
I	28 (4.7)	62 (6.1)	
II	332 (55.2)	613 (60.7)	
III	226 (37.6)	321 (31.8)	
IV	15 (2.5)	14 (1.4)	

Notes: Data are presented as n (%) unless otherwise indicated. IQR=25%-75% interquartile range. Standardized differences were calculated by dividing the differences in means or proportions between the 2 groups by their pooled standard deviations. An absolute standardized difference of >0.1 indicates imbalance.^27^

### Time Interval Variance Overlay Plots

**Figure 1.**
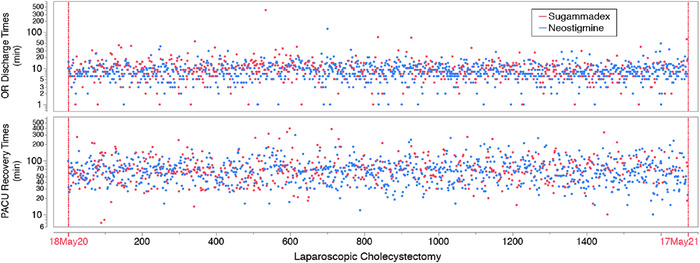
**Overlay plots of operating room (OR) discharge (upper panel) and postanesthesia care unit (PACU) recovery (lower panel) time intervals expressed in minutes (min) (logarithmic scale) for the two neuromuscular blocking reversal agents—sugammadex in red and neostigmine in blue—during the 1-year study period. Autocorrelation values for the overlay plots were 0.004 for OR discharge times and 0.03 for PACU recovery times.** (For readers of the print publication, a color version of this figure is available online at https://doi.org/10.31486/toj.22.0064.)

The time interval variances for the two reversal agents are expressed in overlay plots in Figure 1. The time interval variances in OR discharge times (upper panel) and in PACU recovery times (lower panel) appear to be consistent throughout the study period. Autocorrelation values for the overlay plots were 0.004 for OR discharge times and 0.03 for PACU recovery times.

### OR Discharge and PACU Recovery Boxplots and Cumulative Distribution Plots

**Figure 2.**
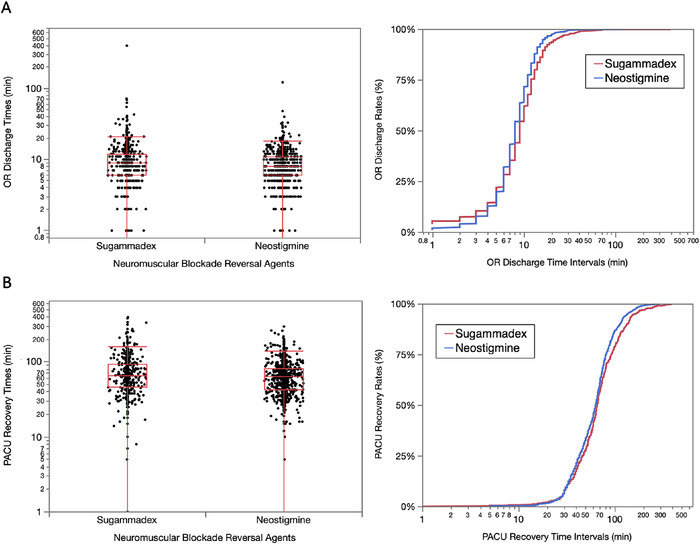
**Boxplots and cumulative distribution plots of operating room (OR) discharge and postanesthesia care unit (PACU) recovery time intervals expressed in minutes (min) (logarithmic scale) for the two neuromuscular blocking reversal agents during the 1-year study period. (A) Arithmetic medians for OR discharge times were 9 [interquartile range (IQR), 6-12] minutes for patients receiving sugammadex and 8 [IQR, 6-11] minutes for patients receiving neostigmine (chi-square = 11.8, *P* = 0.0006). (B) Arithmetic medians for PACU recovery times were 66 [IQR, 46.5-93] minutes for patients receiving sugammadex and 63.5 [IQR, 43-81.3] minutes for patients receiving neostigmine (chi-square = 6.8, *P* = 0.0090).** (For readers of the print publication, a color version of this figure is available online at https://doi.org/10.31486/toj.22.0064.)

OR discharge and PACU recovery times for the two neuromuscular blocking reversal agents are shown in Figure 2. Time intervals for the boxplots are expressed in logarithmic format to improve visual clarity of the data sets.

The geometric means for OR discharge times were 9.1 minutes for patients administered sugammadex and 8.0 minutes for patients administered neostigmine. The arithmetic means for OR discharge times in patients administered sugammadex were 10.9 (95% CI, 10-11.8) minutes vs 8.9 (95% CI, 8.2-9.7) minutes in patients administered neostigmine. Arithmetic medians for OR discharge times were 9 [IQR, 6-12] minutes in patients administered sugammadex and 8 [IQR, 6-11] minutes in patients administered neostigmine (Figure 2A, left panel).

The OR discharge times for the reversal agents are also expressed as cumulative distribution plots in logarithmic format to improve visual clarity of the plot (Figure 2A, right panel). A discernable percentage difference in the discharge slopes for the reversal agents begins to develop at the 6-minute time interval, with discharge percentages higher for patients who received neostigmine vs patients who received sugammadex.

The geometric means for PACU recovery times were 65.5 minutes for sugammadex and 60.8 minutes for neostigmine. The arithmetic means for PACU recovery times in patients administered sugammadex were 77.6 (95% CI, 74.1-81.1) minutes vs 68.6 (95% CI, 65.9-71.3) minutes in patients administered neostigmine. Arithmetic medians for PACU recovery times were 66 [IQR, 46.5-93] minutes in patients administered sugammadex and 63.5 [IQR, 43-81.3] minutes in patients administered neostigmine (Figure 2B, left panel).

The PACU recovery times for the two reversal agents are also expressed as cumulative distribution plots in logarithmic format (Figure 2B, right panel). A discernable percentage difference in the recovery slopes for the reversal agents begins to develop at the 33-minute recovery time interval, with neostigmine recovery percentiles higher compared to sugammadex recovery percentiles in subsequent time measurements.

### OR Discharge and PACU Recovery Equivalence Tests

**Figure 3.**
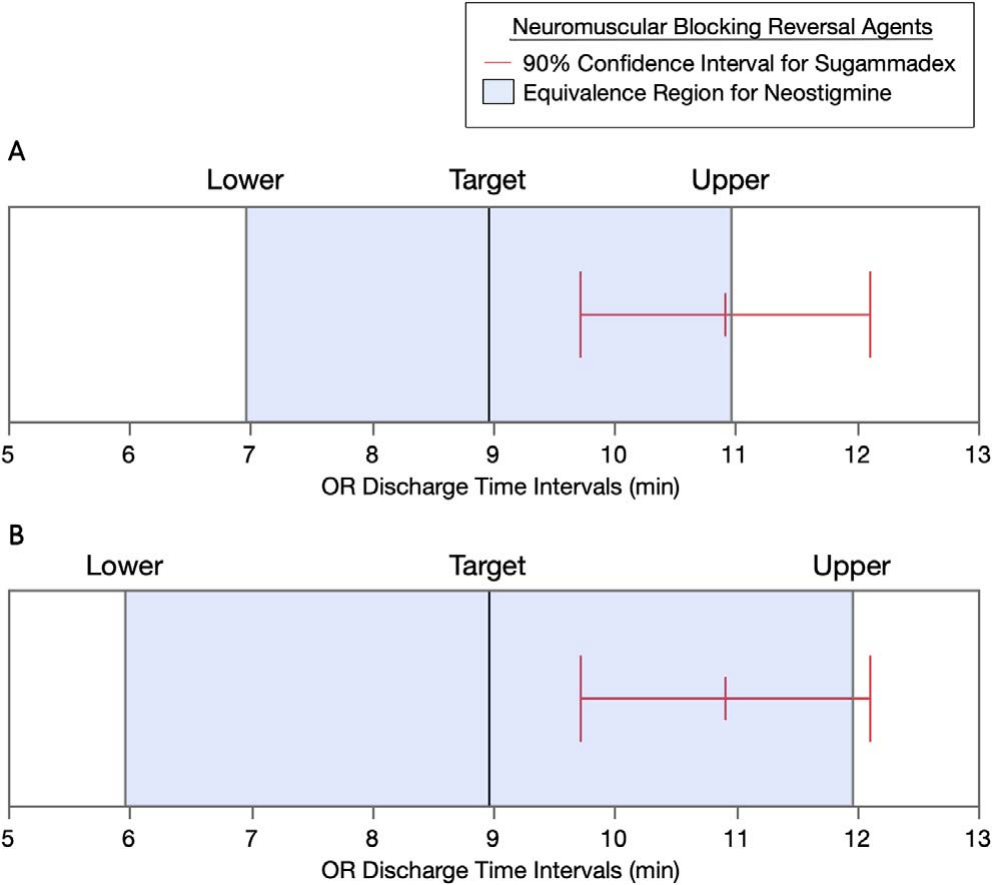
**Equivalence testing of sugammadex to neostigmine by operating room (OR) discharge times. (A) With a 2-minute (min) time interval range and an alpha level = 0.05, the means difference = 1.98, SE 0.77 (90% CI, 0.7-3.2). The upper threshold t-ratio = –0.03, (*P* = 0.4885), the lower threshold t-ratio = 5.2 (*P*<0.0001), and the maximum *P* value of both tests is 0.4885. The 90% CI of sugammadex is not within the target range for neostigmine. Sugammadex is not equivalent to neostigmine under these target range conditions.^22^ (B) With a 3-minute time interval range and an alpha level = 0.05, the means difference = 1.98, SE 0.77 (90% CI, 0.7-3.2). The upper threshold t-ratio = –1.3 (*P* = 0.0912), the lower threshold t-ratio = 6.5 (*P*<0.0001), and the maximum *P* value of both tests is 0.0912. The 90% CI of sugammadex is not within the target range for neostigmine. Sugammadex is not equivalent to neostigmine under these target range conditions.^22^** (For readers of the print publication, a color version of this figure is available online at https://doi.org/10.31486/toj.22.0064.)

**Figure 4.**
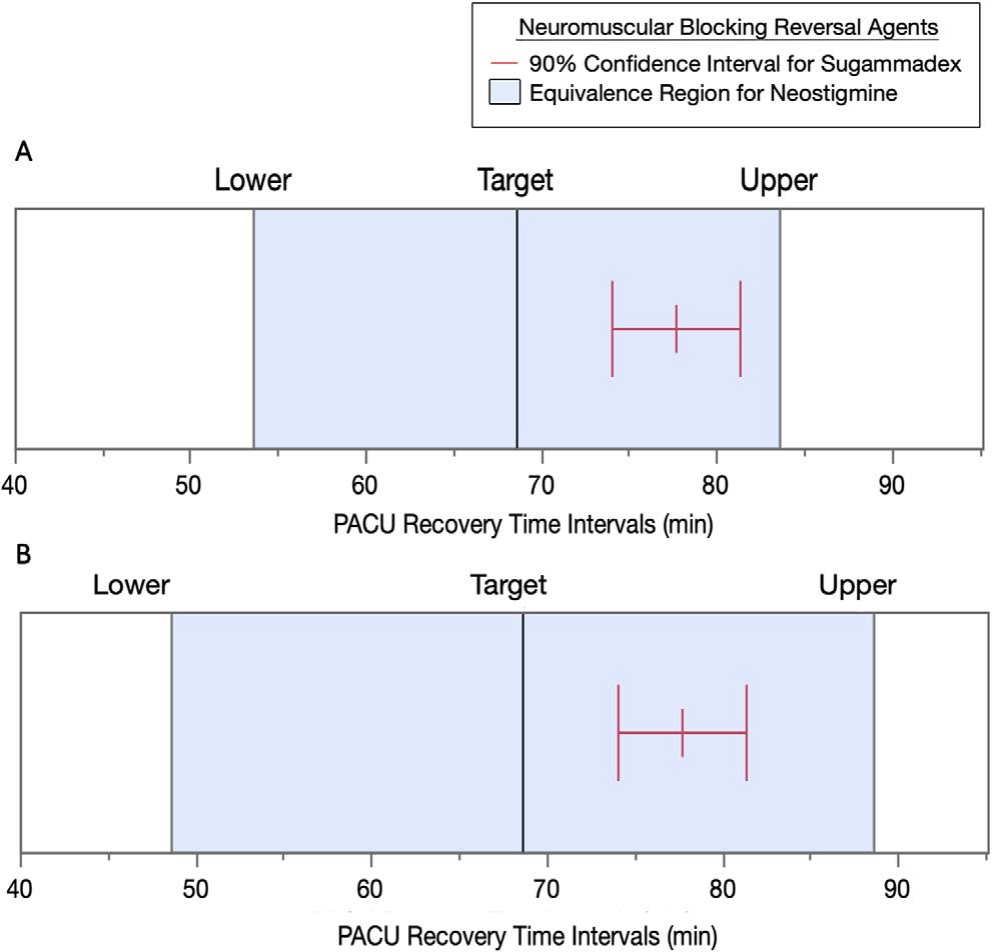
**Equivalence testing of sugammadex to neostigmine by postanesthesia care unit (PACU) recovery times. (A) With a 15-minute (min) time interval range and an alpha level = 0.05, the means difference = 9.1, SE 2.5 (90% CI, 4.9-13.2). The upper threshold t-ratio = –2.3 (*P* = <0.0001), the lower threshold t-ratio = 9.6 (*P*<0.0001), and the maximum *P* value of both tests is <0.0001. The 90% CI of sugammadex is within the target range for neostigmine. Sugammadex is equivalent to neostigmine under these target range conditions.^22^ (B) With a 20-minute time interval range and an alpha level = 0.05, the means difference = 9.1, SE 2.5 (90% CI, 4.9-13.2). The upper threshold t-ratio = –4.3 (*P*<0.0001), the lower threshold t-ratio = 11.6 (*P*<0.0001), and the maximum *P* value of both tests is <0.0001. The 90% CI of sugammadex is within the target range for neostigmine. Sugammadex is equivalent to neostigmine under these target range conditions.^22^** (For readers of the print publication, a color version of this figure is available online at https://doi.org/10.31486/toj.22.0064.)

The results of equivalence testing for the two reversal agents by recovery periods are shown in Figures 3 and 4. When the delta (δ) time intervals for equivalence in OR discharge times were set for either ±2 minutes (Figure 3A) or ±3 minutes (Figure 3B) of the target value for neostigmine, the 90% CI for sugammadex was not within the target range. When the δ time intervals for equivalence in PACU recovery times were set for either ±15 minutes (Figure 4A) or ±20 minutes (Figure 4B) of the target value for neostigmine, the 90% CI for sugammadex was within the target range.

## DISCUSSION

Neuromuscular blocking agents are used to facilitate endotracheal intubation and improve surgical conditions.^23-26^ Reversal of neuromuscular blockade is frequently required through the use of acetylcholinesterase inhibitors, most commonly neostigmine, which has been in continuous use since the 1950s.^30,31^ However in December 2015, the US Food and Drug Administration approved sugammadex for use in reversing neuromuscular blockade induced by either rocuronium or vecuronium in adults.^32^ Sugammadex is a modified gamma-cyclodextrin ring with a central core to bind either rocuronium or vecuronium. Based upon the speed in reversing neuromuscular block when compared to neostigmine,^5,8^ additional studies proposed that routine use of the modified gamma-cyclodextrin could provide additional OR discharge and PACU recovery time savings.^6,7^ Although randomized controlled trials should evenly distribute known and unknown factors between groups, thereby reducing the potential for confounding, randomized controlled studies still have limitations.^19^ Randomized controlled trials do not represent patients from the general population because of restrictive eligibility criteria and can suffer from lack of external validity.^33-35^ Eventually, the clinical effectiveness of any new medication needs examination under real-world, non-Hawthorne-effect conditions.^18-21^ In this study, all patients undergoing laparoscopic cholecystectomy under general clinical practice conditions during a 1-year period were entered into this study. Other than the 61 patients who were excluded because of neostigmine-induced residual neuromuscular blockade requiring sugammadex, the participants represented a general population of patients requiring this surgical procedure. Although treatment bias for sugammadex was observed in the setting of higher ASA physical status classifications, recovery time intervals did not improve with sugammadex under these practice conditions.

The variability of the time intervals following neuromuscular blocking reversal therapy during the 1-year study period was examined with overlay plots. Overlay plots provide a framework to visually detect changes that may represent the introduction of an unknown confounder during the study period. Autocorrelation statistics were also performed and detected no cyclical patterns, suggesting a stable clinical care period.

The data were also displayed in cumulative distribution plots to allow comparative visual analysis throughout the two time periods. Although the initial OR discharge and PACU recovery time percentiles for sugammadex were higher than those observed for neostigmine, the benefits were soon lost at the 6-minute OR discharge time interval and at the 33-minute PACU recovery time interval, with the majority of recovery percentiles in both graphs favoring neostigmine. Initial studies suggested that sugammadex could provide shorter OR discharge times and shorter PACU recovery times.^9,14^ John and colleagues reported improved OR discharge times with sugammadex, an interval mean decrease of 2.7 (95% CI, 0.2-5.2) minutes.^9^ Carron and colleagues reported an interval mean decrease of 22 (95% CI, 15-30) minutes in OR discharge times, as well as an improvement in PACU recovery times (approximately 17 minutes) with sugammadex.^14^ In our study, we did not observe these OR discharge or PACU recovery time savings using equivalence testing methods,^22^ as both 90% CIs for sugammadex were above the targets set for neostigmine. The differences between these studies and ours are unknown, although inclusion and exclusion criteria possibly played a role.^9,14^ Another reason could be that a Hawthorne effect^18-21^ occurred in these studies.^9,14^ Nevertheless, our data analyses under conditions of general clinical practice suggest that sugammadex does not provide improvement in the time intervals of interest following laparoscopic cholecystectomy.

### Strengths and Limitations

One limitation of this study is that the patients did not undergo preprocedural randomization that would support unbiased allocation of treatment. Although the baseline ASA physical status classifications between the two reversal groups were unbalanced,^27^ the two recovery times revealed no clinically important differences. Moreover, clinical judgment is an important component of individualized clinical care, which is limited in randomized controlled clinical trials.^33-35^ The randomness of patterns observed in the overlay plots for both time intervals of interest were stable across the study period, which suggests that external forces were not introduced during this clinical care period. An additional strength of this study is no influence of a Hawthorne effect which is now recognized as a problem with randomized controlled trials because of changes in the behavior of health care personnel during experimental conditions.^21^ Another strength is the use of cumulative distribution plots to allow comparative assessment of the reversal agents across individual recovery time percentiles rather than through assessments summarized with geometric or arithmetic means and medians. Finally, these analyses are from a group of patients undergoing clinical care under generalized practice conditions.

## CONCLUSION

These results suggest no benefit of sugammadex when used as a primary neuromuscular blocking reversal agent to improve either OR discharge or PACU recovery times when compared to neostigmine following laparoscopic cholecystectomy.

## Notes

*This article meets the Accreditation Council for Graduate Medical Education and the American Board of Medical Specialties Maintenance of Certification competencies for Patient Care, Medical Knowledge, and Practice-Based Learning and Improvement.*
